# Ranging patterns and factors associated with movement in free‐roaming domestic dogs in urban Malawi

**DOI:** 10.1002/ece3.8498

**Published:** 2022-01-27

**Authors:** María De la Puente‐Arévalo, Paolo Motta, Salome Dürr, Charlotte Warembourg, Christopher Nikola, Jordana Burdon‐Bailey, Dagmar Mayer, Frederic Lohr, Andy D. Gibson, Patrick Chikungwa, Julius Chulu, Luke Gamble, Neil E. Anderson, Barend M deC. Bronsvoort, Richard J. Mellanby, Stella Mazeri

**Affiliations:** ^1^ Independent Consultant Madrid Spain; ^2^ European Commission for the Control of Foot‐and‐Mouth Disease FAO Rome Italy; ^3^ Veterinary Public Health Institute, Vetsuisse Faculty University of Bern Bern Switzerland; ^4^ Mission Rabies Cranborne UK; ^5^ Department of Animal Health and Livestock Development Lilongwe Malawi; ^6^ The Roslin Institute and The Royal (Dick) School of Veterinary Studies, Easter Bush Veterinary Centre University of Edinburgh Midlothian UK; ^7^ The Epidemiology, Economics and Risk Assessment Group, The Roslin Institute and The Royal (Dick) School of Veterinary Studies, Easter Bush Veterinary Centre The University of Edinburgh Midlothian UK; ^8^ Division of Veterinary Clinical Studies, Hospital for Small Animals, The Roslin Institute and The Royal (Dick) School of Veterinary Studies, Easter Bush Veterinary Centre The University of Edinburgh Midlothian UK

**Keywords:** domestic dog, home range, Malawi, rabies, roaming behavior, utilization distribution

## Abstract

Rabies is a neglected zoonotic disease that causes around 59,000 deaths per year globally. In Africa, rabies virus is mostly maintained in populations of free‐roaming domestic dogs (FRDD) that are predominantly owned. Characterizing the roaming behavior of FRDD can provide relevant information to understand disease spread and inform prevention and control interventions. To estimate the home range (HR) of FRDD and identify predictors of HR size, we studied 168 dogs in seven different areas of Blantyre city, Malawi, tracking them with GPS collars for 1–4 days. The median core HR (HR50) of FRDD in Blantyre city was 0.2 ha (range: 0.08–3.95), while the median extended HR (HR95) was 2.14 ha (range: 0.52–23.19). Multivariable linear regression models were built to identify predictors of HR size. Males presented larger HR95 than females. Dogs living in houses with a higher number of adults had smaller HR95, while those living in houses with higher number of children had larger HR95. Animals that received products of animal origin in their diets had larger HR95, and only in the case of females, animals living in low‐income areas had larger HR50 and HR95. In contrast, whether male dogs were castrated or not was not found to be associated with HR size. The results of this study may help inform rabies control and prevention interventions in Blantyre city, such as designing risk‐based surveillance activities or rabies vaccination campaigns targeting certain FRDD subpopulations. Our findings can also be used in rabies awareness campaigns, particularly to illustrate the close relationship between children and their dogs.

## Graphical abstract

In Africa, rabies virus is mostly maintained in populations of free‐roaming domestic dogs (FRDD) that are predominantly owned. Characterizing the roaming behavior of FRDD can provide relevant information to understand disease spread and inform prevention and control interventions. In our study, we tracked with GPS collars 168 FRDD in Blantyre city, Malawi, to estimate their home range and identify predictors of HR size.

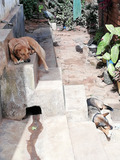

## 1 INTRODUCTION

Dogs have been long‐time companions of human beings, but in spite of all positive aspects, this coexistence facilitates sharing of multiple parasites, viruses, and bacteria between the two species. Rabies is the disease transmitted from dogs to humans with the highest fatality rate, with examples of human survival after clinical presentation of the disease being extremely rare in the literature (Gilbert et al., 2012). Rabies is caused by an RNA virus of the *Rhabdoviridae* family which can affect all mammals. Despite this wide range of hosts, the most common transmission pathway to humans is from domestic dogs. Dog‐mediated rabies, often transmitted through the bite of a rabid dog, is responsible for more than 99% of human rabies deaths (WHO, 2021). Rabies is estimated to cause around 59,000 human deaths annually around the world (Hampson et al., 2015) and Malawi has one of the highest rabies death rates per capita of any country, with over three deaths per 100,000 persons annually (Hampson et al., 2015). In 2012, the Queen Elisabeth Central Hospital of Blantyre city, in Southern Malawi, reported that in only 3 months (September–November 2011), five children died of rabies. Until that date, the estimated incidence of rabies recorded by the institution was approximately five cases per year (Depani et al., 2012). The number of cases of rabies in Blantyre city and in the district has decreased since 2015, following a successful annual mass canine vaccination program carried out by the non‐governmental organization Mission Rabies (Zimmer et al., 2018).

Despite this success, rabies remains a public health threat and dog welfare issue in Blantyre city (Hampson et al., 2015), demanding frequent vaccination campaigns. The dog population of Blantyre city has been estimated to be 45,526 (95% CI 45,147–45,906), with 97.1% of dogs considered to be owned and a human:dog ratio of 18.1:1 (Gibson et al., 2016). Although most of the dogs in Blantyre city are owned, many of them are not continually restrained inside the household/compound, and are allowed to roam freely during part of the day or night or even at all times. In Malawi, as in other African countries, rabies virus is maintained in populations of owned free‐roaming domestic dogs (FRDD) (Conan et al., 2015). Therefore, characterizing dog roaming patterns can provide relevant information to understand disease spread and inform disease mitigation interventions. Defining how far from their household dogs roam can help estimate potential number of contacts with other animals, which is important for understanding disease spread (Hudson et al., 2019). A deeper knowledge of the factors affecting roaming behavior will lead to identify high‐risk individuals. This information would help to refine recommendations for rabies vaccination targeting these specific dogs (Warembourg, Fournié, et al., 2021). Prioritization of animals could reduce vaccination costs in low‐income countries where veterinary services do not have enough budget to carry out dog vaccination campaigns (Lembo et al., 2010). Increasing the knowledge of rabies in communities is essential for disease prevention, reaching in particular the most vulnerable groups, which are not often receiving crucial information (Tiwari et al., 2021). A better understanding of dog roaming patterns can be of help to define risk factors for rabies exposure and therefore to identify these most exposed groups toward which awareness campaigns should be addressed.

The home range (HR) and the utilization distribution (UD) are two concepts that are broadly used in ecology to describe the roaming patterns of animals. The HR can be defined as the area an animal commonly uses for normal activities, such as foraging, hunting, and breeding (Burt, 1943). The UD is an estimation of the relative frequencies with which an animal uses the various areas of its HR (Benhamou, 2011). Various studies have looked at the roaming behavior of FRDD by estimating their HR, using different data collection approaches. However, in the last decades, the use of GPS loggers has been the method of choice to collect position data necessary to study the animals’ roaming behavior (Table A1). The statistical methods selected in different studies to estimate HR differ, with Minimum Convex Polygon (MCP) (Garde et al., 2016; Meek, 1999; Melo et al., 2020; Pérez et al., 2018; Sparkes et al., 2014; Vaniscotte et al., 2011) and Biased Random Bridge (BRB) (Dürr & Ward, 2014; Dürr & Ward, 2014; Hudson et al., 2017; Molloy et al., 2017; Muinde et al., 2021; Warembourg, Wera, et al., 2021) being the most commonly used estimators. The study of FRDD roaming behavior using GPS devices has taken place in different regions around the world (Table A1), and with the exception of a few of them in Chile, Peru, Brazil, Kenya, Guatemala, Indonesia, and Uganda (Melo et al., 2020; Muinde et al., 2021; Pérez et al., 2018; Raynor et al., 2020; Warembourg, Wera, et al., 2021), these studies took place in rural areas.

There are considerable differences in the HR size estimates published and therefore, it is difficult to extrapolate the available results to a new study site. Much of this research has also analyzed whether factors such as age (Dürr et al., 2017; McDonald et al., 2020; Molloy et al., 2017; Muinde et al., 2021; Pérez et al., 2018; Warembourg, Wera, et al., 2021), sex (Dürr et al., 2017; Hudson et al., 2017; McDonald et al., 2020; Melo et al., 2020; Molloy et al., 2017; Muinde et al., 2021; Sparkes et al., 2014; Van Kesteren & Torgerson, 2013; Warembourg, Wera, et al., 2021), body condition (McDonald et al., 2020; Molloy et al., 2017; Pérez et al., 2018; Warembourg, Wera, et al., 2021), or neutering status (Dürr et al., 2017; Garde et al., 2016; Melo et al., 2020; Molloy et al., 2017; Sparkes et al., 2014) can be predictors of HR size, and studies have not always reached the same conclusions (Table A1). Additional research in new locations and socio‐economic contexts can contribute to define which are the factors that can predict HR size in each setting. To the best of our knowledge, this is the first study tracking FRDD in a city in Southern Africa.

The aim of this study was to characterize movement patterns of FRDD in Blantyre city, Malawi, by estimating their HR and UD. We also aimed to identify the key factors associated with HR size in the study site, considering predictors of space use identified in previous studies and other potentially relevant variables. Additionally, we compared the two most commonly used HR estimators to assess if the results obtained varied depending on the method used. The study results can be used to inform rabies surveillance and rabies control interventions.

## 2 MATERIALS AND METHODS

### 2.1 Ethics statement

This study involved placing GPS collars on 223 dogs for a period of 4–5 days. The collars were placed only after obtaining informed consent by the owner or a person responsible for the animals. The study proposal was reviewed and approved by the Human and the Veterinary Ethical Review Committees of the University of Edinburgh (HERC_352_19; VERC_82_19). Approval was also obtained from the Ministry of Agriculture, Irrigation and Water Development, Department of Animal Health and Livestock Development, Malawi (Ref. No 15/10/32 a).

### 2.2 Study site

**FIGURE 1:**
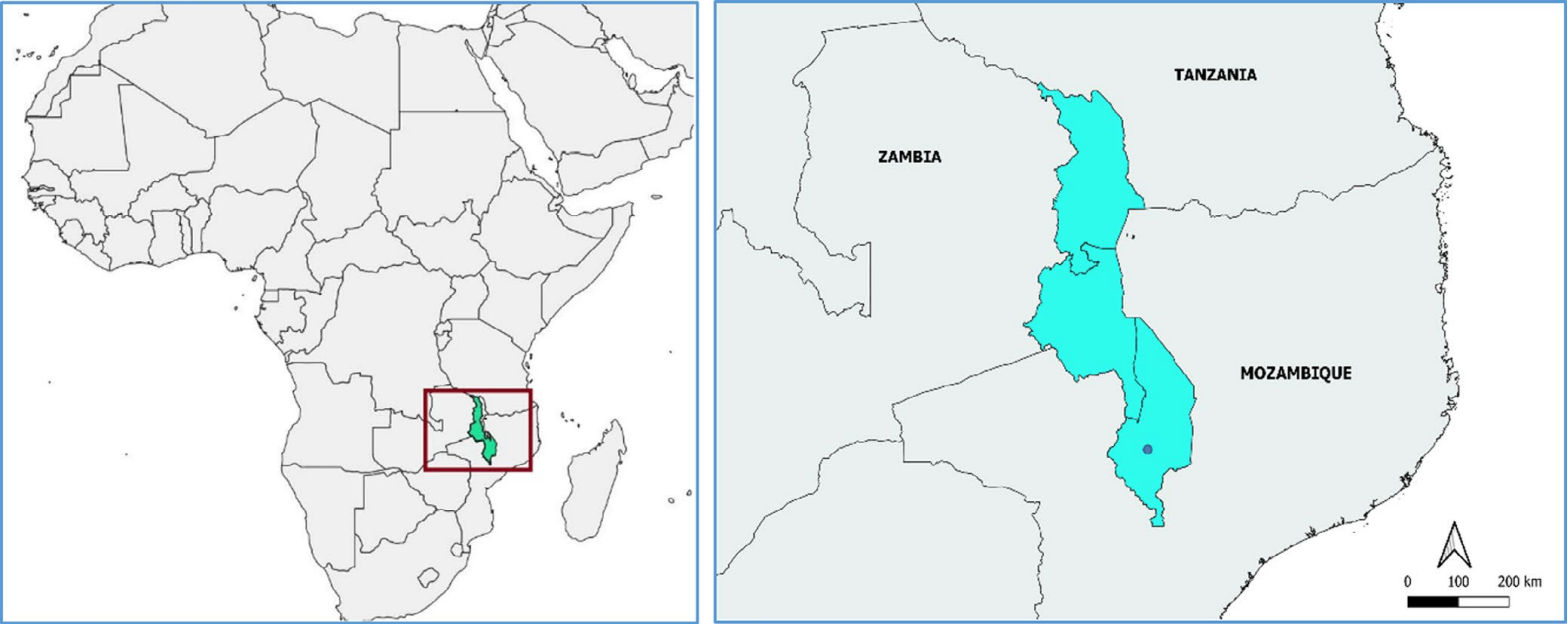
Map of Africa showing the location of Malawi (country shaded in blue); Map of Malawi showing the location of Blantyre city (purple dot). The maps were created with QGIS (https://qgis.org) using maps from Natural Earth (www.naturalearthdata.com) and GADM (https://gadm.org/)

Data collection took place in Blantyre city, Malawi, from June 27th until August 9th 2019. Blantyre city, in the Southern Region, with 800,000 inhabitants (National Statistical Office, 2018), is the second biggest city in Malawi and the country's most important commercial hub (Figure 1). The city is divided into 25 administrative wards where, in most of the cases, urbanization is poorly planned and access to basic services is limited (United Nations Statistics Division, 2021).

**FIGURE 2:**
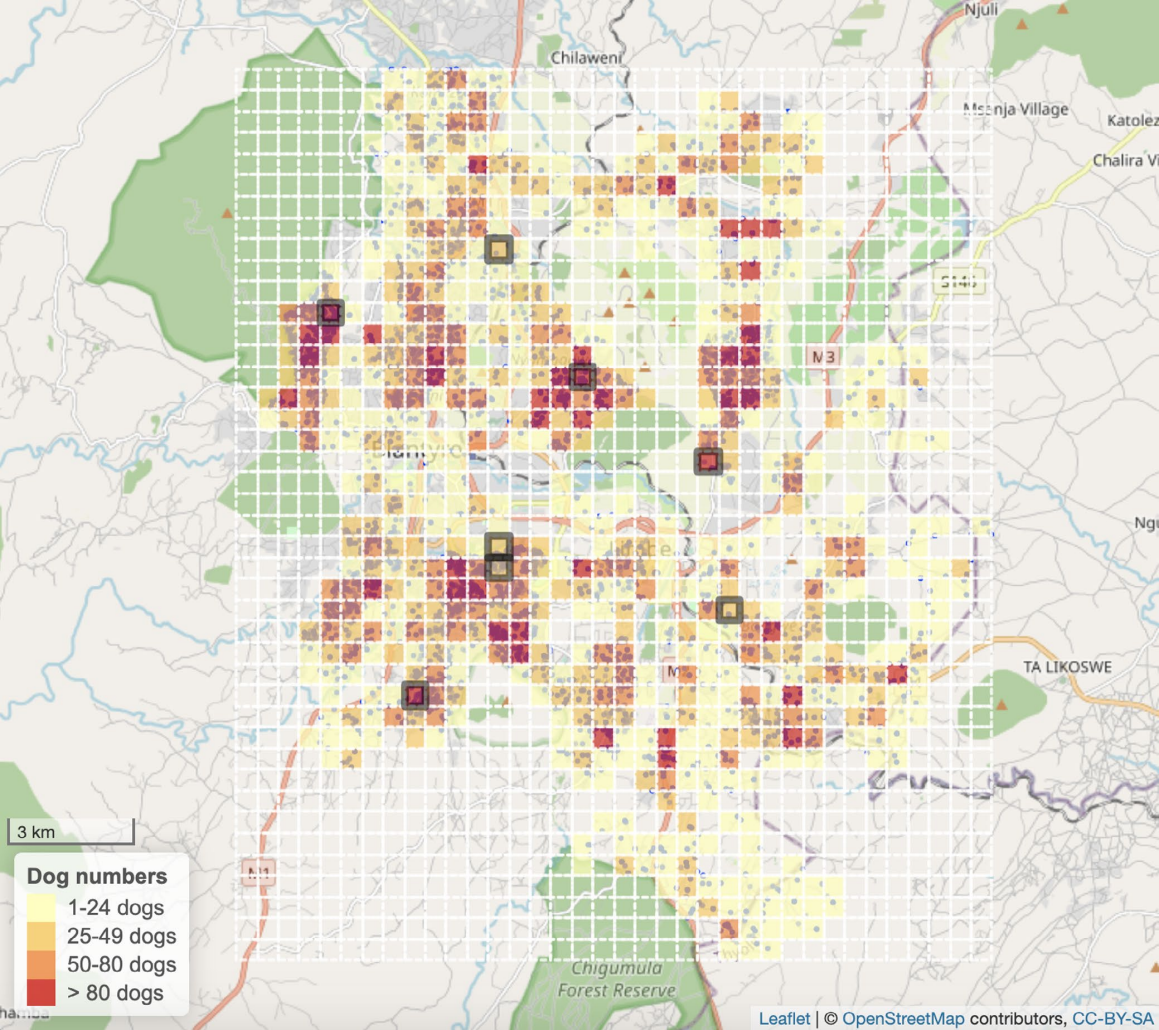
Map showing the division of Blantyre city in 500 m × 500 m areas and the seven areas selected to place the collars (highlighted with a gray border). The areas are colored based on the number of dogs found during the 2017 Mission Rabies vaccination campaign. One of the areas is formed by two adjacent squares and is therefore larger than the other ones. The map was created with R package leaflet (Cheng et al., 2019) using tiles sourced from OpenStreetMap

In order to select the areas where the GPS collars would be placed on dogs, Blantyre city was divided in 500 m × 500 m squares. During the 2017 Mission Rabies door‐to‐door dog rabies vaccination campaign in Blantyre city, the location of all dogs seen was recorded using the Worldwide Veterinary Service (WVS) smartphone data collection application (Gibson et al., 2018; Sánchez‐Soriano et al., 2020). These data were used in our study to extract the number of dogs seen in each square and classify them in terms of dog density (low density = <50 dogs per square, medium density = 50–80 dogs per square, and high density >80 dogs per square). Seven areas were selected randomly among low and high dog population density squares (three and four areas, respectively). In one of the areas, Area 3, as the number of FRDD found was very low, collars were also placed in part of an adjacent 500 m × 500 m square with similar characteristics. As a result, Area 3 was larger than the other ones (1,000 m × 500 m) (Figure 2). Three of the areas selected were low‐income areas (LIA) (Maoulidi, 2012). LIA visited were characterized by high density of houses, with households having access to a pit latrine toilet and, in general, lacking a fence around them.

### 2.3 GPS collars and data collection

Forty six GPS collars, which were donated by their producer, Trakz Ltd (https://trakz.io/), were used in the study. Each collar weighed less than 40 g (Trakz Labs Ltd, 2021) and consisted of a GPS unit within a sheath adjustable to the dog's neck. GPS units were configured and the data collection schedule was set through Trakz application, where the devices had been registered in advance: The time between two GPS fixes was set at 1 min when the animal was moving and at 60 min when the dog was not active to ensure a longer duration of the battery. Movement was detected through the device's inbuilt accelerometer. Data recorded by the GPS units (geographical coordinates and time for each GPS fix) and information on the accuracy of each GPS fix in meters (used to inform HR calculation) could be accessed through the backend of Trakz application. Together with the backend, a mobile phone application allowed the team to track the dogs in real time, if needed (e.g., to recover the collar).

A team of two to three data collectors went to the selected areas, with at least one veterinarian and a dog handler always present. Each square visited to place the GPS collars was accessed from one edge and every household with dogs found along the path followed was included in the study if the following conditions were met: (a) the owner or one responsible person for the dog/s was at home and was at least 18 years old; (b) consent to be part of the study was given; (c) the dog/s were allowed to roam freely during all or part of the day or night; and (d) dog/s could be restrained for collar placement. The team stopped visiting households once all available tracking devices were placed. The surface covered in each square varied depending on the number of FRDD present at the time of deployment.

In every household that took part in the study, the owner or responsible person for the animals was asked to respond to a questionnaire aimed at collecting information related to the respondent, the household visited, and the dogs owned, including general information about each animal, its origin, and management practices ([Link], [Link], [Link]). Questions were read in English or Chichewa, depending on the participant's preference, and answers were recorded in the form prepared in the WVS smartphone data collection application (Gibson et al., 2018). Some of the information was collected through visual inspection of the animals, such as estimation of the reproductive status or the body condition based on the World Small Animal Veterinary Association (WSAVA) score chart (WSAVA, 2013). The WVS application was also used to ensure that the households selected were always inside the 500 m × 500 m area. This was possible through the application's “Pathtracker” functionality (Gibson et al., 2018), which allows the user to visualize the path followed against the delimitated study area.

The collars were left on the dogs for 4–5 days. Four days was the maximum duration of the GPS units´ battery, based on the data collection schedule and data recording parameters used. During the visit to retrieve the collars, the owner or carer of the dog/s was asked if the dog/s had been taken somewhere during the collaring period, particularly by car. This information was used to clean the dataset prior to data analysis.

### 2.4 Data cleaning

The datasets from each GPS unit were downloaded as csv files from the backend of Trakz application. Similarly, the datasets collected through the questionnaire‐based survey using the WVS application were downloaded as csv files. The two datasets were then prepared for data analysis and analyzed in R Statistical Software (R Core Team, 2020). Datasets from the following GPS units were not considered for the analysis: Units that did not work (*n* = 15), units that worked for less than 24 h or that registered a low number of fixes (less than 78, which corresponded to the 5 percent quantile of number of fixes per collar) (*n* = 31), and units that belonged to dogs that were taken to another location during the study period (*n* = 4).

**FIGURE A1:**
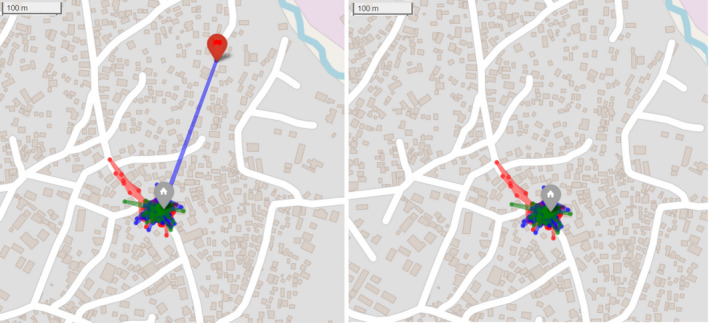
Example of fixes recorded for a dog before and after the cleaning process. The figure shows an outlier (left) considered an error and eliminated from the dataset (right). The map was created with R package leaflet (Cheng et al., 2019) using tiles sourced from OpenStreetMap

Some GPS units registered isolated fixes that were located too distant from the previous and successive fixes to consider feasible that the movement between these points was achieved by the dog walking or running. These fixes were therefore assumed to be errors and were removed to clean the datasets. The method used to identify and remove these outliers was the one described by Dürr and Ward (2014) based on the maximum speed that a dog can reach. This method assumes that a community dog is very unlikely to run at speeds of more than 20 km/h during a 1 min period. Therefore, the speed between consecutive fixes was calculated and the consecutive GPS fixes resulting in speeds of >20 km/h were automatically removed (221 fixes). This method proved appropriate when the dog was moving and therefore fixes were recorded every minute. However, when the dog was inactive and therefore fixes were recorded every 60 min, some outliers were not detected by this method. Therefore, in addition, a final visual inspection of all plotted datasets was made and these remaining errors were manually removed (38 fixes) (Figure A1).

### 2.5 Data analysis

#### 2.5.1 Home range estimations

The core home range (HR50), which is defined as the area where 50% of the activities of an animal take place (Powell, 2000), and the extended home range (HR95), which is the area where the animal carries out 95% of its activities, were estimated using the “adehabitatLT” and “adehabitatHR” R packages (Calenge, 2006). In our study, two methods were used to estimate the HR: one as primary choice and a second one to compare the results obtained when a different estimator is used. The BRB method (Benhamou, 2011; Benhamou & Cornélis, 2010) was the first choice in our study due to its realistic approach and its suitability for a dataset with GPS fixes recorded at an irregular frequency (Dürr & Ward, 2014). This method allows to estimate the UD, and using a specific function of the “adehabitatHR” R package (Calenge, 2006), HR was then derived for the 50% and 95% isopleths. There are three parameters that are required when the BRB is used: A maximum time (Tmax) between two consecutive GPS fixes above which the segment between them is not considered in the UD computation (Dürr & Ward, 2014). Tmax was set to 6 min as in previous studies where the GPS units were set to record fixes every minute (Dürr et al., 2017; Dürr & Ward, 2014; Hudson et al., 2017; Molloy et al., 2017). Another parameter required by the BRB method is Hmin, which describes the distance between the real location of the GPS device and the location registered (Molloy et al., 2017). Hmin was estimated for each device as the mean of the accuracy information in meters registered for every GPS fix. The mean accuracy of GPS fixes registered by each collar was approximately 12 m (mean of the means by collar = 12.66; range = 6.91–20.25). The last parameter, Lmin, defines the minimum distance between fixes below which it is considered that the animal is resting (Molloy et al., 2017) because the BRB method does not consider the resting time to estimate the HR (Benhamou, 2011). Lmin was set for each collar as twice the Hmin, assuming that if two consecutive fixes are separated by a distance below two times the mean accuracy of each GPS device, the animal might have moved but is still resting.

The MCP (Hayne, 1949; Mohr, 1947) was selected in our study as a second method to estimate HR50 and HR95 and build additional models. This method was chosen for having been widely used to estimate home range size and study ranging behavior of multiple mammal species, and is therefore a better method to compare our findings with others (Nilsen et al., 2008). The function removes 50% and 5% of the fixes farthest away from the centroid of the HR to estimate HR50 and HR95, respectively (Calenge, 2006). The agreement between the results obtained by the two methods (BRB and MCP) was evaluated through the Bland–Altman analysis (Altman & Bland, 1983). This method creates a scatter plot where the Y‐axis represents the difference of the HR estimated by the two methods for each dog (i.e., HR50 BRB‐HR50 MCP) and the X‐axis shows the mean of the HR estimated by the two methods for each pair of observations (i.e., (HR50 BRB + HR50 MCP)/2). Three parallel lines to the X‐axis are represented in the plot: One line corresponding to the mean of the differences between the paired HR; two lines defining an interval within which 95% of the differences between the HR estimations by the two methods fall (Giavarina, 2015).

#### 2.5.2 Multivariable linear regression models

Twelve multivariable linear regression models were built to explain if variation in HR size could be attributed to variation in the explanatory variables included in the respective models. To build these models, four different dependent variables were used (i.e., HR50 and HR95 estimated by the two different methods, BRB and MCP) and different subsets of the sample population were considered (i.e., total, males, and females). In the model with only a subset of the population, independent variables that only were relevant for males or females were included (e.g., neuter status for males, pregnancy for females) in addition to the variables tested with the dataset of the total population. The following steps were followed to build and evaluate each of the models, after log‐transforming the dependent variable data because they were not normally distributed:

(a) Twenty‐five independent variables (Table A2) were considered to build an equivalent number of univariable linear regression models in order to select those variables to be included in the multivariable linear regression model; (b) Explanatory variables whose coefficient had a *p*‐value below .25 were pre‐selected for inclusion in the multivariable regression model; (c) Correlations or associations were checked between those pairs of pre‐selected explanatory variables where a relationship was considered plausible. When a correlation or an association was identified between a pair of variables, only one of the pair was considered for inclusion in the model; (d) The multivariable linear regression model was built with the final selection of independent variables; (e) In order to select the combination of variables that best fitted the model, a backward stepwise approach was followed. The lowest Akaike's Information Criterion (AIC) was used to select the best model; (f) Residuals of the model were visualized to confirm they were distributed randomly; (g) Outliers that were over‐proportionally influential data points were identified computing Cook's distance (Cook, 1975) for the best‐fitted (lowest AIC) model 1 (i.e., HR50 estimated by the BRB with all dogs included) and model 3 (i.e., HR95 estimated by the BRB with all dogs included). As a consequence, two outliers were removed from the dataset for the final analysis with all models whose dependent variable was HR50 (*n* = 166) and three for those modeling HR95 (*n* = 165); (h) Steps (a)–(f) were repeated for the 12 models without these dogs; and (i) The twelve final models were run again considering plausible interactions between the explanatory variables. The new models considering interactions were selected if they showed a better fit (lower AIC).

## 3 RESULTS

### 3.1 Datasets

A minimum of 26 and a maximum of 36 animals were collared in each of the seven sites, representing 80% of the total FRDD seen along the paths walked (Table 1).

**TABLE 1:** Sampling areas and GPS collars placed

Sampling areas	Area in Blantyre city	Classification of the area	Estimated number of dogs^a^	Number of FRDD seen	Number of FRDD collared	Number of FRDD missed
Area 1	Makheta. Nkolkoti	LIA^b^	83	36	36	0
Area 2	Chilobwe	Non‐LIA	103	38	35	3
Area 3	Queen Elizabeth Central Hospital & Chitawira^c^	Non‐LIA	36 + 48	54	34	20
Area 4	Michiru	Non‐LIA	119	55	33	22
Area 5	Chirimba	Non‐LIA	26	35	30	5
Area 6	Bangwe	LIA	38	34	29	5
Area 7	Ndirande	LIA	175	27	26	1
TOTAL			628	279	223	56

Note

The estimated number of dogs per area is based on the number of dogs seen in the 2017 Mission Rabies door‐to‐door rabies vaccination campaign. Number of FRDD collared in each sampling area. Missed FRDD are those that could not be collared. The number of FRDD seen in an area is the addition of the dogs collared and the dogs that, despite being allowed to roam, could not be included in the study (e.g., not collared because they were too aggressive).

^a^ Number of dogs recorded during the 2017 Mission Rabies door‐to‐door dog rabies vaccination campaign (Sánchez‐Soriano et al., 2020).

^b^ Low‐income area

^c^ In Area 3, as the number of FRDD was very low, collars were also placed in part of an adjacent 500 m × 500 m square with similar characteristics.

Of 223 collar placements, 50 were excluded during the data cleaning process, therefore 173 dogs whose movements were registered for 1–4 days were used for data analysis. From the data collected by these units, 259 (0.19%) fixes considered errors were removed from a total of 132,854 fixes registered for these 173 dogs. Once cleaned, the number of fixes per dog was on average 766 (range: 89–1575).

Finally, the code used to estimate the HR50 and HR95 using the BRB method failed for five datasets that were removed, resulting in 168 dogs to be considered in the linear regression models. For better comparison of the results, these five dogs were not considered in any of the models, regardless of the estimator used.

### 3.2 Population structure and dog management habits

The 168 dogs included in the study belonged to 102 households from the seven different sampling areas selected. The median number of adults per household was 3 (range: 1–10) and the median number of children (individuals under 18 years of age) was also 3 (range: 0–9). The median number of dogs per household was 2 (range: 1–7).

Table 2 summarizes the dog population structure. In relation to the management practices, there was a significant difference (*p* < .001) between male (*n* = 30; 36% of the males) and female dogs (*n* = 1; 0.01% of the females) that were neutered. When owners were asked about their primary purpose for owning a dog, 90% of them (150 dogs) indicated protection of the house. Furthermore, 95% of the dogs (*n* = 159) were always allowed to roam freely outside of the property during the night, while only 43% (*n* = 72) of them were always allowed to roam freely during the day.

Most of the respondents answered that they specifically prepared food for their dogs (*n* = 163, 97% of the dogs), with “nsima,” a porridge prepared with maize flour very common in the Malawian diet, being the most common complementary food provided to the animals. Additionally, for 54% of the animals, (*n* = 90) owners reported that this complementary food also included products of animal origin (PAO) (i.e., bones, fish, dog food, and meat). Seventy‐four percent (*n* = 67) of dogs that received PAO lived in areas classified as non‐LIA. Finally, half of the dogs had a shelter built for them and only four dogs were not taken for anti‐parasite treatment (“dipping”).

**TABLE 2:** Population structure

Population characteristics	Number of dogs	Proportion of dogs (%)
Sex		
Male	83	49
Female	85	51
Breed		
Local	117	70
Mixed	51	30
Size		
Small	1	0.5
Medium	165	98
Large	2	1.5
Age		
1–3 years	93	56
3–6 years	61	36
>6 years	14	8
Body condition		
Thin (bcs = 1–3)	17^a^	10
Normal (bcs = 4–5)	149	89
Obese (bcs = 6–9)	2	1
Pregnant females		
Yes	23	27
No	62	73
Females lactating puppies		
Yes	15	18
No	70	82
Females in heat		
Yes	7	8
No	78	92
Females that had puppies at least once		
Yes	56	66
No	29	34

Note

The first five categories in the table refer to characteristics of the total study sample (*n* = 168); the last four categories refer to characteristics of the female dog subset of the population (*n* = 85)

^a^ 16 females; 1 male.

### 3.3 Human‐mediated movements

Fifty‐nine dogs out of 168 (35%), were born in the house they belonged to, while 88 (52.5%) were obtained from a neighbor, 19 (11.5%) were bought from a roadside seller, and 2 (1%) were found on the street by the owners. The origin of the dogs that were bought or were a gift from a neighbor was the same ward as where they currently lived in most of the cases (*n* = 58; 66%), but some of the dogs came from another ward in Blantyre city (*n* = 24; 27%) or another district in the country (*n* = 6; 7%). It was not possible to know the exact origin of the dogs bought from a roadside seller; however, for 53% of these dogs (*n* = 10), the interviewees reported they bought the animal from a roadside seller who was in the same ward; for 37% of the dogs (*n* = 7), the roadside seller was in another ward; and 10% of the dogs were bought from a roadside seller in another district of Malawi (*n* = 2). Although this information was not specifically collected during this study, it is generally accepted that in Blantyre city, dogs that are bought or received as gifts are usually acquired when they are still puppies.

### 3.4 Home range sizes

**FIGURE 3:**
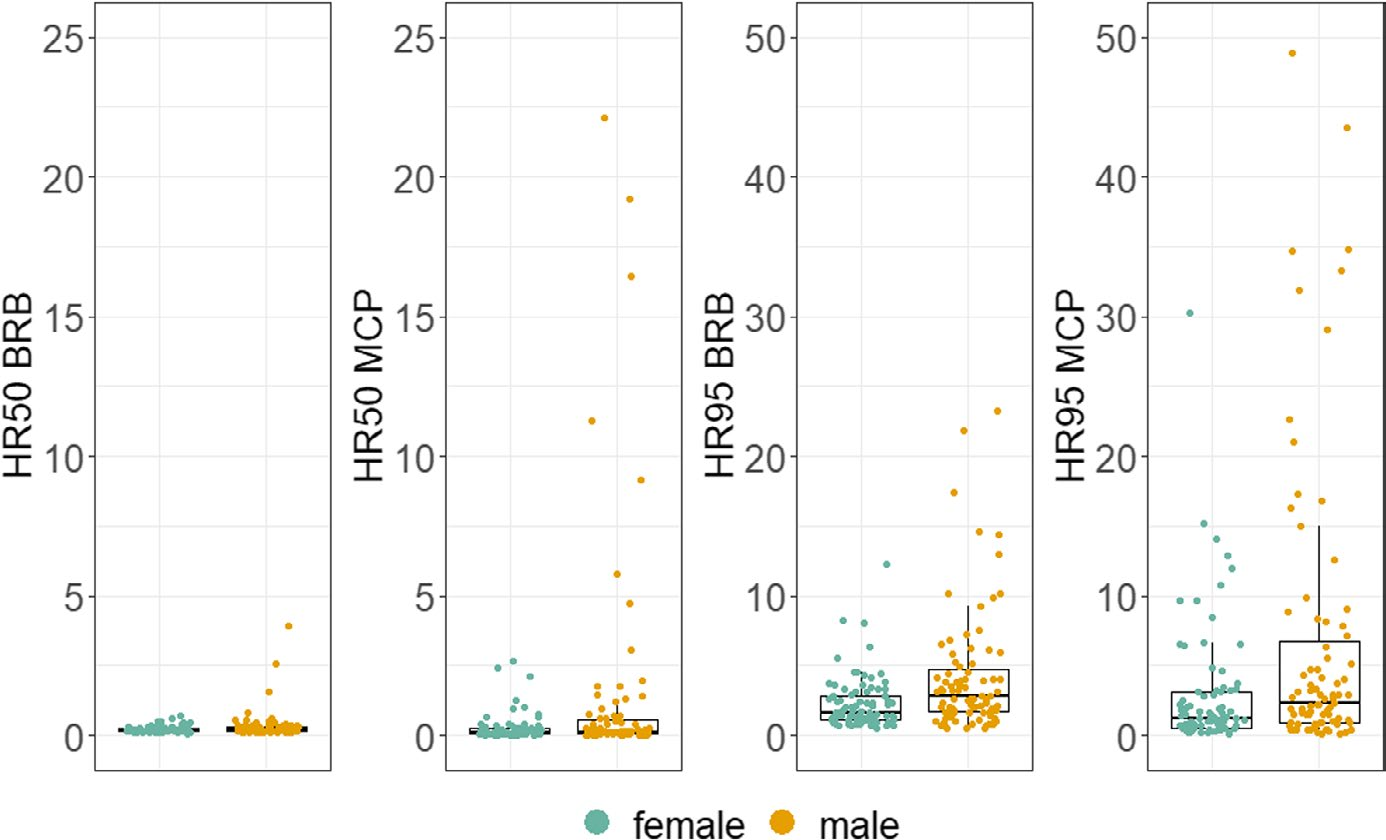
Boxplots of the HR50 and HR95 values (in ha) estimated using the BRB method and the MCP for the dog sample divided by sex. Scales used for HR50 and HR95 are different

**FIGURE 4:**
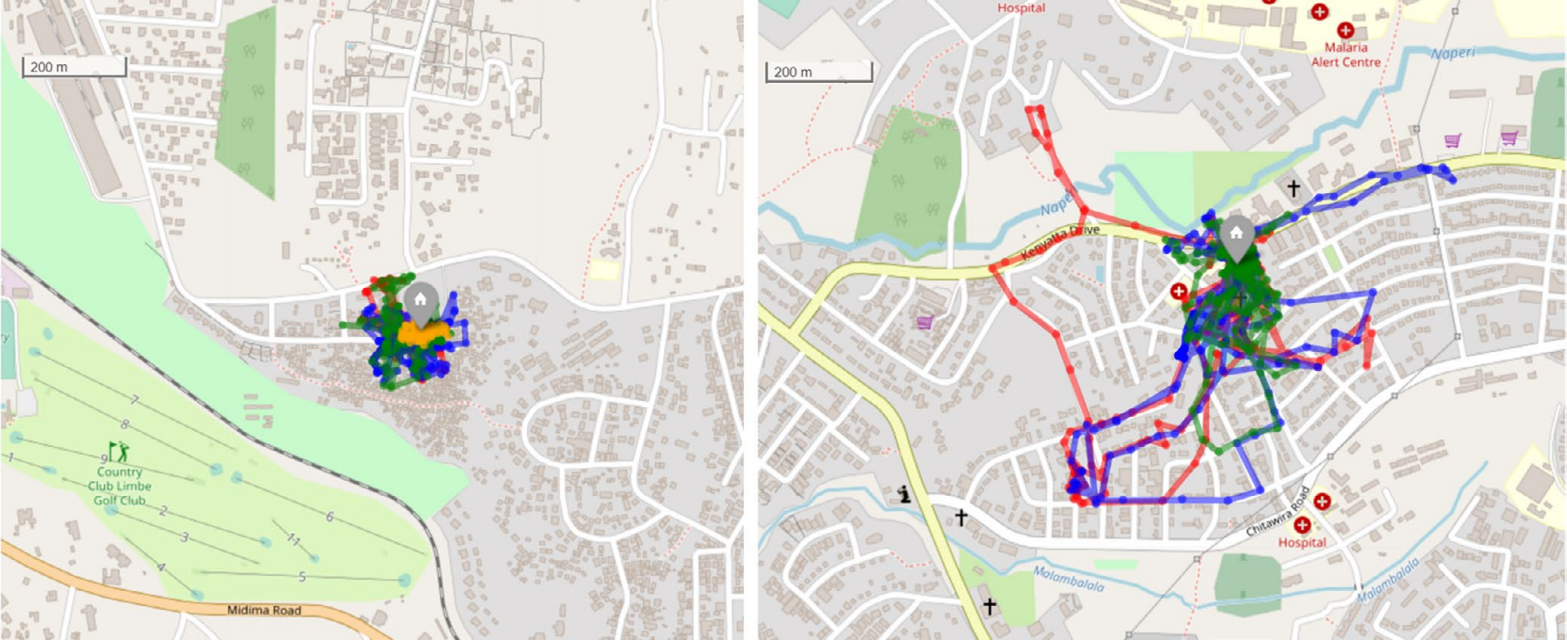
Pathways followed by two example dogs during a maximum period of 4 days (day 1 red, day 2 blue, day 3 green, and day 4 yellow) The left plot corresponds to a dog with HR95 close to the median (HR95 = 2.15 ha), while the dog to the right has one of the largest HR95 (HR95 14.62 ha). The maps were created with R package leaflet (Cheng et al., 2019) using tiles sourced from OpenStreetMap

**FIGURE A2:**
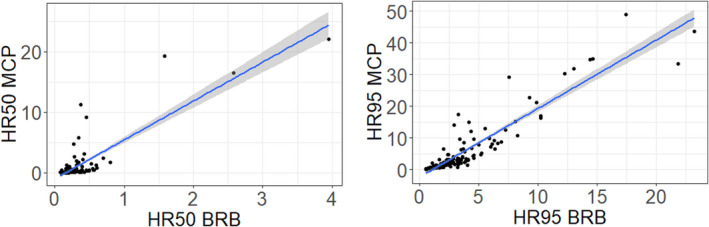
Scatter plots presenting the HR50 (left) and HR95 (right) estimations made using the BRB and the MCP methods. The blue line is the linear regression line between the estimations by the two methods and the gray area shows the 95% CI

**FIGURE A3:**
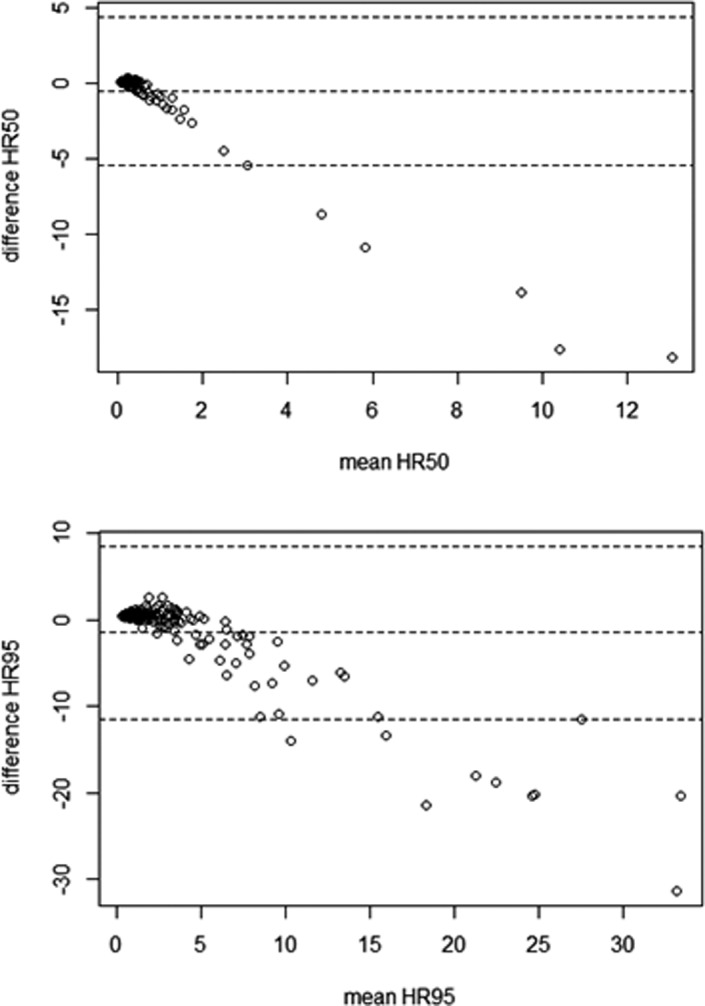
Bland–Altman plot for HR50 and HR95 estimations by the BRB and MCP methods. Y‐axis represents the difference of the HR50 and HR95 estimated by BRB and MCP methods. X‐axis shows the mean of the HR50 and HR95 estimated by the two methods. The middle line represents the mean of the differences between the paired HR50 and HR95 estimations. The other two lines define the agreement limits

The HR50 estimated by the BRB method ranged between 0.08 and 3.95 ha (mean = 0.28, median = 0.2), while the HR95 ranged between 0.52 and 23.19 ha (mean = 3.28, median = 2.14). When the MCP was used, HR50 and HR95 mean values were higher, medians were lower, and HR50 and HR95 value ranges were wider than with the BRB method (HR50: mean = 0.81, median = 0.12, range = 0.01–22.14; HR95: mean = 4.8, median = 1.66, range = 0.15–54.32) (Figure 3). The comparison of the HR estimations obtained by the two methods revealed substantial discrepancies and the Bland–Altman analysis showed that the agreement between methods was lower for higher HR values (Figures A2 and A3). Figure 4 shows two example dogs whose activities occur mainly around their households, with forays to more distant locations.

### 3.5 Predictors of home range size

**FIGURE 5:**
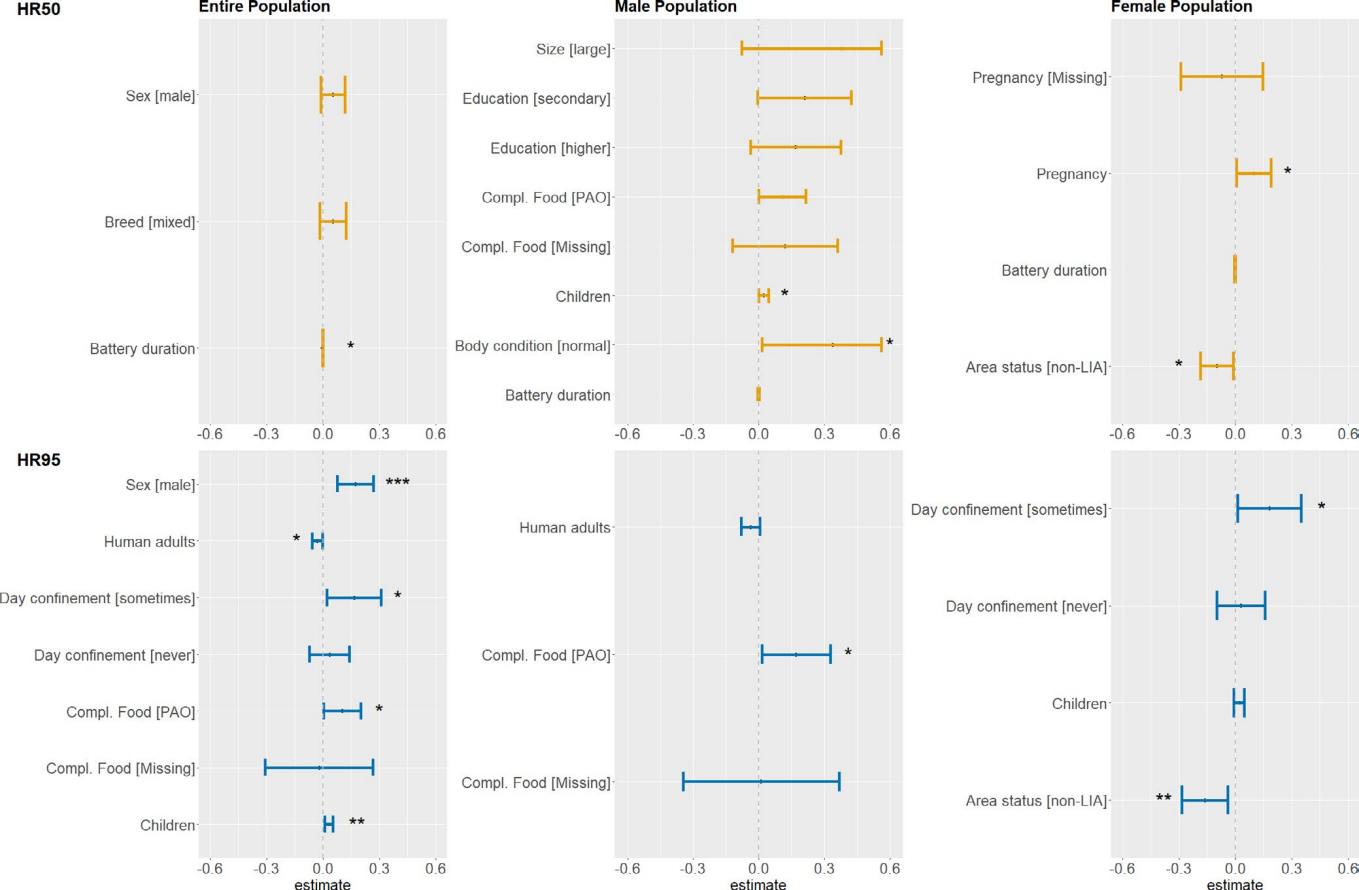
Plot showing the coefficient estimates and 95% CI for each explanatory variable included in the final models (lowest AIC) that used the BRB to estimate HR. Yellow and blue are used to distinguish models with HR50 and HR95 as dependent variable, respectively. The star indicates a significant association between the coefficient estimated for an explanatory variable and HR size (*p <.05; **p <.001; ***p <.001). The reference levels for the categorical values can be found in Table A2

The final models (based on the lowest AIC) included between one to six independent variables, of which some were significantly associated with the dependent variable (*p* value < .05) (Figure 5). When comparing each pair of best‐fit models (i.e., HR estimated with the BRB method versus HR estimated using the MCP), the outputs presented substantial differences (Table A3). In what follows, only the results obtained with the six models that used the BRB method for HR calculation are summarized and later discussed in this document.

Sex and pregnancy were intrinsic factors to the animals that had a significant association with HR size: Males were found to have significantly larger HR95 than females, while pregnant females had a larger HR50 than non‐pregnant females. The households and area where the dogs were living were also associated with HR size. Dogs belonging to households with a higher number of children had a larger HR95, although this effect was not observed in the models with the subpopulations of male and female dogs. HR50 size was significantly associated with the number of children in the household in the male‐only model. In contrast, dogs belonging to households with a higher number of adults had a significantly smaller HR95. Again, this association was not found in the male‐ or female‐only models. In addition, HR50 and HR95 were estimated to be smaller for female dogs living in non‐LIA compared to females living in LIA. Some management practices also showed an association with HR size. Dogs fed with complementary food including PAO had significantly larger HR95 than those that only received nsima and/or other food of plant origin. This effect was also found in the model built only with males, but not with female dogs. Dogs that were sometimes free during the day had significantly larger HR95 compared to dogs that were never free during the day. This was also observed in the female‐only model but not in the male‐only subpopulation. Finally, the GPS device battery duration was only found to be significantly negatively associated with HR size in one of the twelve models (Results in Table A3).

## 4 DISCUSSION

Rabies represents a leading zoonotic and public health threat in Malawi where, as in other African countries, it is maintained in populations of owned FRDD (Conan et al., 2015). We estimated the HR50 and HR95 of 168 FRDD in seven different areas of Blantyre city, Malawi, and identified factors associated with HR size to better understand how rabies and other dog diseases can spread.

Dogs in Blantyre city showed smaller roaming areas than dogs in previous studies where GPS devices were used and the BRB was the HR estimator selected (Dürr et al., 2017; Dürr & Ward, 2014; Hudson et al., 2017; Molloy et al., 2017; Muinde et al., 2021; Warembourg, Wera, et al., 2021). Our smaller HR could be partially explained by the lower Hmin used (Mean Hmin = 12.66) compared to that of the studies mentioned (Hmin = 17–20 depending on the study), as HR sizes are sensitive to Hmin value (Dürr & Ward, 2014). Hmin describes the distance between the real location of the GPS device and the location registered (Molloy et al., 2017) and therefore its value depends on the accuracy of the device used. This might pose a challenge to compare results of studies that have used the BRB as an estimator, but using different GPS loggers and therefore different Hmin values. HR sizes in our study were smaller than those in any of these previous studies, independently on whether they were carried out in an urban or rural setting. Melo et al. (2020) claimed that dogs in urban settings in Brazil have smaller HR compared to rural settings, while Warembourg, Wera, et al. (2021) also observed significantly smaller HR for urban dogs in Guatemala. To the best of our knowledge, studies on roaming behavior in rural Malawi do not exist to be able to identify any difference between urban and rural contexts within the country. There are other factors that could partially explain the smaller HR of FRDD in Blantyre city, such as the overall good body condition of the animals in our sample population, the climatic conditions, or differences in dog management practices. However, these potential reasons explaining our smaller HR would need to be researched further.

Our results presented substantial differences between HR50 and HR95 estimations by the MCP and the BRB, with greater ranges for the results obtained by the MCP method. Furthermore, the Bland–Altman plot showed that for higher HR values, the difference between the two methods tends to increase, which is probably associated with the higher sensitivity of MCP to extreme values in comparison with the BRB method (Dürr & Ward, 2014). Nilsen et al. (2008) investigated to what extent the home range estimator used affected the biological interpretation of the results in comparative studies, concluding that the unpredictable bias in the MCP method might severely affect the results when comparisons are done within species or populations. Our results support these conclusions and illustrate the importance of selecting the most appropriate estimator considering the type of population studied, study done, and data available. Considering these differences based on the estimator used will be important if the results of dog roaming behavior studies are used to inform certain disease control interventions, such as defining the size of the ring vaccination area.

We identified different intrinsic and extrinsic animal factors associated with HR size. In Blantyre city, male dogs had larger HR95 than female dogs. This aligns with previous studies that also found this difference by sex (Dürr et al., 2017; Molloy et al., 2017; Sparkes et al., 2014; Warembourg, Wera, et al., 2021). However, other researchers did not find significant differences between male and female HR size (Hudson et al., 2017; McDonald et al., 2020; Van Kesteren & Torgerson, 2013; Wilson‐Aggarwal, Goodwin, Swan, et al., 2021). We found differences in the effect of other explanatory variables on HR size between male and female dogs, which might be related to different management practices or motivations to roam by sex.

We did not find any significant difference between the HR of neutered and entire male dogs; however, we could not evaluate this factor in females as there was only one neutered female in our dataset. Similarly, no effect of the neuter status on HR was observed in previous studies conducted in Chile (Garde et al., 2016) and Brazil (Melo et al., 2020) while other studies in northern Australia (Dürr et al., 2017; Molloy et al., 2017) found that neutered male dogs roam less than non‐neutered ones.

The fact that pregnant females had larger HR50 than non‐pregnant females could be explained by an increased roaming behavior close to the household to search for food as the intake needs during the pregnancy augment. However, these results can only be considered preliminary as the classification of the animals between pregnant or not was based on the answers provided by the owners during the interviews and on our own visual inspection, which might have inaccurately detected the pregnant status of some female dogs.

Breed and body condition were not found to be predictors of HR size in the different models where they were included, which is consistent with the findings of previous studies (Dürr et al., 2017; Molloy et al., 2017; Pérez et al., 2018; Warembourg, Fournié, et al., 2021; Warembourg, Wera, et al., 2021; Wilson‐Aggarwal, Goodwin, Swan, et al., 2021). One exception is model 5, where underweight males were found to have significantly smaller HR50. Warembourg, Wera, et al. (2021) in Guatemala and Pérez et al. (2018) in Chile found that dogs with poor body condition had smaller HR. However, our results in the male‐only model might not be representative of Blantyre male dog population, as in our sample only 2 males of 83 did not have good body condition. Age was not found to affect HR size either, which aligns with previous studies (Dürr et al., 2017; McDonald et al., 2020; Molloy et al., 2017; Pérez et al., 2018), although some others (Warembourg, Wera, et al., 2021; Wilson‐Aggarwal, Goodwin, Swan, et al., 2021) found that younger dogs have smaller HR.

The association between HR95 size and the number of adults and children in the household might suggest differences in interactions affecting dog roaming behavior. Previous studies showed that dog movements are affected by the activities of their owners (Hudson et al., 2017; Maher et al., 2019; Wilson‐Aggarwal, Goodwin, Moundai, et al., 2021). For example, in urban Malawi, children commonly take care of the dogs and are often accompanied by them when they walk or play away from the household.

HR50 and HR95 were significantly smaller for female dogs collared in non‐LIA. This might be associated with different management practices affecting roaming behavior in these areas. Dürr et al. (2017) observed smaller HR in communities with lower FRDD density, although dog density was not identified as a significant predictor in the multivariable analysis. Although we did not quantify the number of FRDD in each area visited, more time was needed to place the GPS collars in non‐LIA than in LIA. In non‐LIA, there were more gated houses, meaning that many dogs were not allowed to roam freely, and therefore suitable animals to be collared were more difficult to find.

The larger HR95 of dogs that received prepared food comprising products of animal origin compared to those that only received food of plant origin might be associated with the different nutrient composition of these diets. The risk of taurine deficiency has been shown to be higher in dogs fed with low‐protein diets (Sanderson et al., 2001) with lethargy being one of the typical clinical manifestations of this deficiency (Fascetti et al., 2003), possibly resulting in a reduced roaming behavior. However, these results can only be considered preliminary, as in our study questions related to the diet received by the dogs were aimed at understanding the level of care provided to the animals and the objective was not to study specifically the composition of the diet or frequency of feeding. Further studies should explore in detail what FRDD eat and analyze micronutrient deficiencies to study potential associations with HR size.

No difference was observed in HR size between dogs that were always free or always restrained during the day. Similarly, Vaniscotte et al. (2011) observed that tethering the dogs during the day did not influence the distance they roamed during the night. However, HR95 was significantly larger for dogs that were sometimes free during the day. This result is difficult to contextualize in the current study because we could not quantify how frequently these dogs were allowed to roam, and if they were allowed to roam at all during the day on the days they carried the GPS collar.

Finally, our results were not conclusive on whether the duration of the GPS battery, and therefore the number of hours the dogs were tracked for, affects HR size. Similarly, Dürr et al. (2017) did not find differences between dogs monitored for 1–4 days and dogs that were tracked for 5–14 days.

The results of our study confirm the importance of investigating FRDD in different environments (Dürr et al., 2017; Warembourg, Wera, et al., 2021) to understand roaming patterns and factors affecting them. The use of two methods to calculate HR allowed us to compare the results of the analysis done with the two sets of HR estimations, highlighting that the HR estimator selected can affect the results obtained in studies aimed at understanding FRDD roaming behavior.

Some of the outcomes of this study can help inform different interventions for disease control in the study setting. In Blantyre city, human‐mediated movements should be considered as a possible pathway of rabies introduction, either because people get a dog from a friend or relative in another ward in Blantyre city (14% of the dogs) or district in the country (3.6% of the dogs), or because the dog is bought from a roadside seller (11.5% of the dogs), in which case, the origin of the animal is more difficult to define. Colombi et al. (2020) developed a model to identify mechanisms for rabies dispersal in Central African Republic and concluded that “the continuous re‐introductions of rabid dogs via human mediated movements are critical in sustaining the disease in the country.” Based on these results, awareness campaigns to vaccinate dogs introduced from other parts of the country where rabies vaccination coverage might not be as high as in Blantyre city, as soon as they are acquired, could be a useful rabies introduction prevention strategy. Regulating roadside selling of puppies would also be an important public health and animal welfare intervention. Estimated FRDD HR size could be used as an input parameter to model disease spread in urban settings in Southern Africa, not only for rabies but also for other infectious diseases, such as echinococcosis. Besides, in the case of a rabies outbreak, if ring vaccination is applied to dogs as a measure to prevent disease spread, the area to cover should account for FRDD HR size estimated in this study. However, decisions on the size of the vaccination ring should also consider that dog roaming behavior could be affected by rabies symptomatology. For example, dogs developing furious rabies, which occurs in less than 50% of the cases (Fekadu & Shaddock, 1984; Jayakumar et al., 1990), may present agitation as one of the clinical signs (OIE, 2008), which might cause that they roam further. Based on their roaming behavior, male dogs in Blantyre city might be considered at higher risk of getting infected and/or transmitting rabies or other zoonotic diseases. Being a male could be a factor to consider in risk‐based surveillance activities, and in the case rabies vaccination of some individuals needs to be prioritized due to a limited number of vaccine doses available. The results of this study could also be used to illustrate the close relationship between children and their dogs, and therefore to raise awareness on the importance of rabies education for children. Finally, the outcomes of this study can also be relevant to support outbreak investigation when the contacts with a rabid dog need to be traced. Although dogs generally transmit rabies during the clinical phase of the disease, rabies can potentially be transmitted before its onset, as the virus can be detected in saliva up to 14 days before clinical symptoms appear (Fekadu, 1988; Fekadu et al., 1982). During the clinical phase of the disease, the normal behavior of the dog is altered and therefore its usual roaming behavior might also change. However, if contact tracing is carried out considering the period before the disease onset, HR estimations could be very useful to define the area within which people should be interviewed.

Our study presented some limitations that need to be taken into consideration. Although we did have access to recent dog population estimates in Blantyre city, a more up‐to‐date dataset on locations of FRDD would have provided an even better sampling frame. Some dogs were not collared because the owners were not at home at the time of the visit or because they were too aggressive or fearful to be managed. Dogs that spend less time accompanied and dogs which fear their own carers might show a different roaming behavior associated with the type of interactions with their owners, which was not captured in our study. Animals were only followed for a maximum of 4 days and comparison with longer periods of observation would be necessary to confirm whether the time they are tracked for has an effect on HR size. There are other factors that could affect HR size that were not considered in our study, such as whether dogs were tracked on days when a market or livestock slaughter was taking place nearby. Muinde et al. (2021) identified in their study in Kenya some sites that are more frequently visited by dogs, such as rubbish dumps. We could expect a similar dog roaming behavior in Blantyre city, being sites where food is available, such as markets, a point of attraction for FRDD. Animals in our study were only monitored during the dry and hot season, but seasonal weather changes could also be a factor affecting HR size. Dürr et al. (2017) and Wilson‐Aggarwal, Goodwin, Moundai, et al. (2021) found in their respective studies in Australia and Chad that FRDD roamed less during the wet season. Tracking dogs in Blantyre city during the wet season would be necessary to identify whether these seasonal differences that could be relevant for disease transmission exist. Finally, the collection of information through visual inspection and interviews presented some limitations in relation to some of the parameters studied (e.g., pregnancy in female dogs).

To improve the understanding of the risks of rabies introduction in urban settings, such as Blantyre city, further studies on the role of human‐mediated dog movements would be advisable. Additional research would also be needed to better understand how the human–dog bond and veterinary interventions, such as sterilization and castration, affect roaming behavior.

In this study, we estimated HR of FRDD and identified several predictors of their roaming behavior applying a methodology that could inform the design of relevant investigations in other urban contexts. The results of our study can help improve an evidence‐based design and monitoring of rabies prevention and control interventions in the specific context of urban Malawi.

## CONFLICT OF INTEREST

The authors declare that they have no competing interests.

## AUTHOR CONTRIBUTIONS

**María De la Puente‐Arévalo:** Conceptualization (equal); data curation (equal); formal analysis (equal); investigation (equal); methodology (equal); visualization (equal); writing – original draft (lead). **Paolo Motta:** Data curation (supporting); visualization (equal); writing – original draft (supporting); writing – review and editing (equal). **Salome Dürr:** Methodology (supporting); writing – original draft (supporting); writing – review and editing (equal). **Charlotte Warembourg:** Methodology (supporting); writing – review and editing (equal). **Christopher Nikola:** Investigation (equal). **Jordana Burdon‐Bailey:** Investigation (supporting); writing – review and editing (equal). **Dagmar Mayer:** Investigation (supporting); writing – review and editing (equal). **Frederic Lohr:** Writing – review and editing (equal). **Andy D. Gibson:** Writing – review and editing (equal). **Patrick Chikungwa:** Writing – review and editing (equal). **Julius Chulu:** Writing – review and editing (equal). **Luke Gamble:** Funding acquisition (equal); writing – review and editing (equal). **Neil E. Anderson:** Supervision (supporting); writing – review and editing (equal). **Barend M deC. Bronsvoort:** Funding acquisition (equal); supervision (supporting); writing – review and editing (equal). **Richard J. Mellanby:** Funding acquisition (equal); supervision (supporting); writing – review and editing (equal). **Stella Mazeri:** Conceptualization (equal); data curation (equal); formal analysis (equal); investigation (equal); methodology (equal); project administration (equal); supervision (lead); visualization (equal); writing – original draft (supporting); writing – review and editing (equal).

## Supporting information

Data S1

Appendix S1

Supplementary material

## Data Availability

All relevant data used for the analyses have been made available as Supplementary Material.

## APPENDIX A

**TABLE A1:** Summary of results from studies that have estimated the HR of FRDD and/or have analyzed predictors of HR size

HR	Data collection methodology	HR estimation method	Factors studied	Effect on HR size	Location of the study	Reference
4 ha (mean)	51.7 observation‐hours over 16 individual days (15 dogs)	Maximum distance from home used as the radius of a circle‐shaped HR	Degree of restraint Size	More time free, bigger HR Bigger dogs, bigger HR	New York City, USA	Rubin and Beck (1982)
0.2–11.1 ha (summer) 0.1–5.7 ha (winter)	120 observation‐hours in two different seasons (9 dogs in summer and 13 in winter)	Plot of dog locations on scale map. The outermost points were connected while accounting for buildings, streets, and other features of the urban landscape before estimating the area	Season Owned vs. unowned Size Sex	Larger HR in summer Owned dogs, smaller HR No effect No effect	New Jersey, USA	Daniels (1983)
1.74 ha (mean)	17.5 observation‐hours during a 7 months period (8 dogs)	Plot of dog locations on scale maps of the study sites. The outermost points were connected while accounting for buildings, streets, and other features of the urban landscape before estimating the area			Berkeley, California, USA	Berman and Dunbar (1983)
Qualitative estimation	Interviews to owners (122 male dogs)	N/A	Surgical sterilization	Decreases roaming behavior	The Netherlands	Maarschalkerweerd et al. (1997)
Qualitative estimation	Interviews to owners (57 male dogs)	N/A	Surgical sterilization	Decreases roaming behavior	California, USA	Neilson et al. (1997)
Non‐disperser dogs: 4.8 ha Disperser dogs: 8.4 ha (mean)	Weekly observations during daylight hours along a 4 years period (86 dogs)	Plot of dog locations on scale map. The outermost points were connected while accounting for buildings and fenced properties before estimating the area	Season Sex Age	Larger HR in late monsoon, smaller in summer Dispersal more common in males For dispersers: Larger HR if they are older than 1 year	West Bengal, India	Pal et al. (1998)
Sedentary dogs: 2.6 ha Wandering dogs: 927 ha (mean)	Radio‐collars. Dogs tracked over five sessions of 18 h (10 dogs)	MCP. Mean core activity areas also estimated (MCP 60% isopleths)			Aboriginal community in Bherwerre Peninsula, Australia	Meek (1999)
Night core areas (including 80% fixes): 50% dogs < 0.16 ha; 5% dogs > 1.19	GPS. One‐night trajectories (96 dogs)	MCP. UD functions estimated by Kernel method (Worton, 1989)			Four villages in Tibet	Vaniscotte et al. (2011)
2.26 ha (mean)	GPS. Dogs tracked between 1.5 and 47 h (37 dogs)	Characteristic hull polygon (CHP) method (Downs & Horner, 2009)	Village Sex	No effect No effect	Alay Valley, Kyrgyzstan	Van Kesteren and Torgerson (2013)
They used activity range (AR): AR males: 68 ha; AR females: 31.67 ha (mean)	GPS. Dogs tracked for 7 days (20 dogs)	MCP	Sex Surgical sterilization	Males have larger AR No effect	Aboriginal island community in Northern Australia	Sparkes et al. (2014)
Core HR: 0.2–0.4 ha; Extended HR: 2.5–5.3 ha (median). Some dogs: 40–104 ha	GPS. Dogs tracked for 1–3 days (69 animals collared)	Estimation of HR and UD by four different methods: MCP, LKDE, BRB, and T‐LoCoH			Six Aboriginal and Torres Strait Islander communities in Northern Australia	Dürr and Ward (2014)
Non‐scavengers: 12.8 ha; Scavengers: 19.8 ha (mean)	Radio‐tracking and observations for a total of 45 days, 3 h/day (19 dogs)	Kernel density estimator	Turtle nest scavengers and non‐scavengers	No effect	Colola Sanctuary, Mexico	Ruiz‐Izaguirre et al. (2015)
65 ha (mean)	GPS collars. Dogs tracked for 3 days (86 male dogs)	MCP	Chemical sterilization Surgical sterilization Season	No effect No effect No effect	Puerto Natales, Chile	Garde et al. (2016)
They defined roaming patterns: stay‐at‐home, roamer, and explorer dogs. Core and extended HR (ha), respectively: 0.3/3.7, 0.4/6, 0.6/9.5 (mean)	GPS. Dogs tracked in two different periods for 15 and 68 days, respectively (46 in 2014 and 29 dogs in 2016)	UD estimated using the BRB method and HR derived from the 50% and 95% isopleths	Sex	No effect	Northern Australia Indigenous communities	Hudson et al. (2017)
Core HR: 0.35 ha. Extended HR: 4.48 ha (median)	GPS. Dogs tracked for 2–16 days (135 dogs)	UD estimated using the BRB method and HR derived from the 50% and 95% isopleths	Sex/Neutering status Season Dog density Age Breed and genetics	The effect of the dog's sex was significantly dependent on the neutering status Larger HR during pre‐ than post‐wet season Higher‐density, larger HR No effect No effect	Eight Aboriginal and Torres Strait Islander communities in Northern Australia	Dürr et al. (2017)
Core HR: 0.27 ha; Extended HR: 3.1 ha (median)	GPS. Dogs tracked for 1–4 days (58 dogs)	UD estimated using the BRB method and HR derived from the 50% and 95% isopleths	Sex Neutering status Body condition Age Household location Hunting use	Males have larger HR Neutered dogs have smaller HR Thinner dogs, larger HR No effect No effect No effect	Four Aboriginal communities in Northern Australia	Molloy et al. (2017)
65 ha (mean)	GPS. Dogs tracked for 3 days (86 male dogs)	MCP	Body condition Age Household location	Thinner dogs have smaller HR No effect No effect	Puerto Natales, Chile	Pérez et al. (2018)
Core HR: 0.013–46 ha; Extended HR: 0.12–370 ha	GPS. Dogs tracked for 4 days to 4 weeks (23 dogs)	T‐LoCoH	Effect of dry water channels, an urban feature of Arequipa	Dry water channels promote movement	Arequipa, Peru	Raynor et al. (2020)
0.04 ha (mean); 0.003 ha (median)	Geo‐referencing of captured/recaptured locations in seven sampling efforts (270 dogs; HR estimated for 54 of them)	MCP	Sex Neutering status Land cover Commercial food outlets	Females have larger HR No effect Clusters where less vegetation and food outlets	Two municipalities in Southeastern Brazil	Melo et al. (2020)
Core HR: 105 ha (mean) Total HR: 1,042 ha (mean)	GPS. Dogs tracked for up to 14 days (150 dogs)	60% kernel density estimates for core HR and 100% MCP for total HR	Sex Age Body condition Settlement Use in hunting activity Household water provision	No effect No effect Thin roam less Differences No effect No effect	Three settlements in Chad	McDonald et al. (2020)
Core HR: 0.4 ha (median) Extended HR: 9.3 ha (median)	GPS. Dogs tracked for 5 days in two different periods: May–June 2017 and June–July 2019 (73 dogs)	BRB	Age (<1 and ≥1 year) Sex/Neutering status Time spent outside Number of fixes Recording period	Older dogs, larger HR95 Castrated males travel less; neutered females travel further No effect No effect No effect	Eight sites Busia county, Kenya	Muinde et al. (2021)
Core HR (median): 0.3 ha Chad; 0.33 ha in Guatemala; 0.30 ha in Indonesia; 0.25 ha in Uganda. Extended HR (median): 7.7 ha in Chad; 5.7 ha in Guatemala; 5.6 ha in Indonesia; 5.7 ha in Uganda	GPS. Dogs tracked for 60 hours in average (773 dogs)	BRB	Sex Age Body condition Role Time dog is allowed to roam Site	Different results depending on the country	Different countries (2–3 locations per country): Chad, Guatemala, Indonesia, Uganda	Warembourg, Wera, et al. (2021), Warembourg, Fournié, et al. (2021)
Core HR: 2 ha (median) Extended HR: 10 ha (median)	GPS. Dogs tracked for up to 14 days (129 dogs)	AKDE (HR also estimated using the MCP and KDE)	Sex Age Body condition Village Owner hunting Water provision	No effect Older dogs, larger HR No effect No effect No effect No effect	Six villages in Ethiopia	Wilson‐Aggarwal, Goodwin, Moundai, et al. (2021), Wilson‐Aggarwal, Goodwin, Swan, et al. (2021)
Dry season: Core HR: 8 ha; Extended HR: 54 ha (median) Wet season: Core HR: 4 ha; Extended HR: 31 ha (median)	GPS. 174 dogs for 37 days (mean) in the dry season; 151 dogs tracked in the wet season	AKDE (HR also estimated using the MCP and KDE)	Sex Body condition Village Owner hunting Time spent around the household Season	HR larger during dry season; differences by village; Dogs belonging to hunting households with larger HR; More time spent close to the household, smaller extended HR.	Six villages in Chad	Wilson‐Aggarwal, Goodwin, Moundai, et al. (2021), Wilson‐Aggarwal, Goodwin, Swan, et al. (2021)

Note

For each study, the table includes information on the estimated HR size and the methodology used to collect the data and to estimate HR. It also includes the factors considered in each study and whether they were found to have an effect or not in HR size. The location of the study is also indicated.

**TABLE A2:** List of independent variables used to build the univariable linear regression models

Variable name	Description	Type	Possible values
Adult dogs	Number of adult dogs (at least 3 months of age) within the household	Numeric	1–4; 6; 7;
Age	Dog age in years	Numeric	0.3–13
Area status	Binomial classification according to the definition of different Blantyre city areas by Maoulidi (2012)	Categorical	**Low‐income area (LIA)**; non‐LIA
Battery duration	Time the battery of the GPS device lasted in hours	Numeric	24.946–97.267
Body condition	Body condition of the animals based on the WSAVA body condition score: thin for dogs with a score between 1 and 3; normal if the score was 4–5; and obese it scored 6 or more.	Categorical	**Thin**; Normal; Obese
Breed	Dog breed: considering Africanis type of dogs as the local breed, and mixed breed refers to all those animals where features of a foreign breed was recognizable (e.g., Labrador, terrier).	Categorical	**Local**; Mixed breed
Cats	Whether there were cats in the household.	Categorical	**No**; Yes
Children	Number of children in the household (under 18)	numeric	0–9
Complementary food	Whether complementary food given to the dog contained products of animal origin (PAO) or only plant‐based products (non‐PAO)	Categorical	**non‐PAO**; PAO; Missing^a^
Day confinement	Confinement status of the dog during the day	Categorical	**Never free**; Sometimes free; Always free;
Delivered	Whether the female dog had ever had puppies	Categorical	**No**; Yes; Male
Education level	Highest level of education among all people living in the household	Categorical	**Primary**; Secondary; Higher
Heat	Whether the female dog was in heat	categorical	**No**; Yes; Male; Missing
Human adults	Number of adults in the household (18 years or more)	Numeric	1–10
Lactation	Whether the female dog was lactating puppies	Categorical	**Yes**; No; Male
Leftovers only	Whether the dog was only fed with leftovers	Categorical	**No**; Yes
Neuter status	Whether the male dog was neutered	Categorical	**No**; Yes
Night confinement	Confinement status of the dog during the night	Categorical	**Never free**; Sometimes free; Always free;
Other animals	Whether there were other animals in the household	Categorical	**No**; Yes
Pregnancy	Whether the female dog was pregnant	Categorical	**No**; Yes; Male; Missing
Puppies	Number of puppies (below 3 months of age) in the household	Numeric	0–3; 5–8; Missing
Rabies vaccines	Number of rabies vaccines received by a dog during its life	Numeric	0–4; 8
Sex	Dog sex	Categorical	**Female**; Male
Shelter	Whether a shelter was provided to the dog	Categorical	**No**; Yes; Missing
Size	Dog size	Categorical	**Small**; Medium; Large

Note

Each variable has been described and the type of variable and possible values for each of them have been included. The reference level for the categorical variables is indicated in bold.

^a^ Missing is a category established for values that are missing in the dataset

**TABLE A3:** Multivariable linear regression models

Model ID	Description of the model	Independent variables included	Independent variables in the final models	Coefficient	95% CI	*p* value	Adjusted R‐squared
Model 1	HR50‐BRB All dogs	Sex, breed, children, area status, battery duration	Sex [male] Breed [mixed breed] **Battery duration**	0.053 0.053 **−0.002**	−0.011 to 0.118 −0.017 to 0.123 −0.004 to <−0.001	.102 .104 .021	0.046
Model 2	HR50‐MCP All dogs	Sex, breed, age, cats	Sex [male] **Age**	0.178 **0.038**	<−0.001 to 0.356 <0.001 to 0.076	.05 .044	0.037
Model 3	HR95‐BRB All dogs	Sex, breed, age, complementary food*, human adults, children, day confinement, battery duration *The model was also run including area status instead of complementary food (area status and complementary food were associated). Area status did not appear in the final model.	**Sex [male]** **Human adults** **Children** **Day confinement [sometimes free]** Day confinement [always free] **Complementary food [PAO]** Complementary food [Missing]	**0.171** −**0.031** **0.030** **0.165** 0.034 **0.101** −0.021	0.076 to 0.267 −0.060 to −0.003 0.007 to 0.053 0.020 to 0.309 −0.072 to 0.140 0.003 to 0.2 −0.307 to 0.266	<.001 .031 .009 .026 .53 .044 .886	0.117
Model 4	HR95‐MCP All dogs	Sex, breed, age, complementary food*, human adults, children, day confinement *The model was also run including area status instead of complementary food (area status and complementary food were associated). Area status did not appear in the final model.	**Sex [male]** Human adults **Children**	**0.214** −0.046 **0.047**	0.049 to 0.378 −0.094 to 0.002 0.008 to 0.086	.011 .062 .019	0.062
Model 5	HR50_BRB Males	Age, breed, size, body condition, children, complementary food, education level, battery duration	Size [large] **Body condition [normal]** **Children** Complementary food [PAO] Complementary food [Missing] Education level [secondary] Education level [higher] Battery duration	0.38 **0.337** **0.023** 0.107 0.119 0.208 0.167 −0.002	−0.079 to 0.84 0.015 to 0.66 <0.001 to 0.045 <−0.001 to 0.215 −0.122 to 0.36 −0.004 to 0.421 −0.039 to 0.375 −0.005 to 0.001	.103 .041 .046 .051 .328 .055 .11 .179	0.105
Model 6	HR50_MCP Males	Age, education level	**Age**	**0.068**	<0.001 to 0.136	.049	0.036
Model 7	HR95_BRB Males	Age, human adults, complementary food	Human adults **Complementary food [PAO]** Complementary food [Missing]	−0.037 **0.171** 0.01	−0.08 to 0.005 0.015 to 0.326 −0.346 to 0.366	.083 .032 .956	0.06
Model 8	HR95_MCP Males	Human adults, complementary food	Human adults **Complementary food [PAO]** Complementary food [Missing]	−0.066 **0.28** 0.213	−0.139 to 0.006 0.015 to 0.544 −0.392 to 0.818	.074 .039 .486	0.059
Model 9	HR50_BRB Females	Pregnancy, day confinement, cats, area status, battery duration	**Pregnancy [yes]** Pregnancy [Missing] **Area status [non‐LIA]** Battery duration	**0.099** −0.071 −**0.098** −0.002	0.007 to 0.191 −0.291 to 0.147 −0.184 to −0.011 −0.004 to <0.001	.035 .516 .027 .089	0.111
Model 10	HR50_MCP Females	Breed, cats, shelter, area status	Cats [yes] **Area status [non‐LIA]**	−0.258 −**0.226**	−0.60 to 0.083 −0.441 to −0.011	.136 .040	0.077
Model 11	HR95‐BRB Females	Delivered, children, day confinement, shelter, area status	Children **Day confinement [sometimes free]** Day confinement [always free] **Area status [non‐LIA]**	0.020 **0.182** 0.031 −**0.163**	−0.007 to 0.048 0.013 to 0.351 −0.10 to 0.16 −0.285 to −0.041	.147 .035 .636 .009	0.165
Model 12	HR95‐MCP Females	Delivered, lactation, children, day confinement, shelter, area status	Delivered [yes] **Area status [non‐LIA]**	0.204 −**0.246**	−0.016 to 0.423 −0.461 to −0.033	.069 .024	0.079

Note

Independent variables included in each of the models and independent variables in the final models (lower AIC). Coefficients that showed a significant association with the dependent variable (*p* value <.05) are highlighted in bold.
