# Supplementary material for: Ranging patterns and factors associated with movement in free‐roaming domestic dogs in urban Malawi
Source: Ecol Evol. 2022 Jan 27;12(1):e8498. doi: 10.1002/ece3.8498 (PMC8794712; doi:10.1002/ece3.8498)
Supplement: Supplementary file 2 — Appendix S1 [file ECE3-12-e8498-s002.pdf]

Questionnaire uploaded in the WVS data collection application

Consent

Hello, my name is Maria de la Puente and I ´m undertaking this research as part of an MSc dissertation project. Thank you for taking part in this study. This study aims to collect information on dog movement in order to better understand the potential for disease transmission. We will put a collar with a GPS on your dog to record where your dog goes during the following 3 or 4 days. We would also like to make you some questions. This survey collects information about dog characteristics and movements. The questionnaire will take between 10 and 15 minutes to complete. The data gathered will be used to understand dog movements and the factors that influence those. This information will help improve rabies and other infectious diseases control and help Mission Rabies vaccination campaigns to better meet the needs of the local community. Be assured that your individual answers will remain anonymous and strictly confidential, only summary results will be published. We need your name and telephone number to contact you to collect the collar, but these data will be destroyed after the study. Your personal data will be handled appropriately and in compliance with data protection legislation in the European Union and will only be shared with and used by Mission Rabies and researchers involved in this study. You can withdraw from the survey and the study at any moment before the 10th of August 2019, when the study in the country will be over (A Malawian telephone number will be facilitated to participants to contact for withdrawing) Do you have any questions? Are you happy to proceed?

- [ ] Consent given
- [ ] Consent not given
- [ ] Adult respondent not home/available
- [ ] Household does not own dogs

Household ID (01,02,etc):

Household GPS location:

Longitud

Latitud

Respondent information

1. Respondent name:
2. Respondent phone number:
3. Respondent gender: ☐ male ☐ female
4. What is your age?
5. How many people, aged 18, or over live in your household (include self)?
6. How many people, aged 17 or under live in your household?
7. What kind of toilet facility does your household have?

- ☐ Flush-sewer system
- ☐ Flush-to pit latrine
- ☐ Pit latrine
- ☐ No facility/bush/field
- ☐ Other: describe
- ☐ No response

8. What is the highest level of education you have achieved?

- ☐ No education
- ☐ Some primary
- ☐ Complete primary
- ☐ Some secondary
- ☐ Complete secondary
- ☐ College education
- ☐ Higher
- ☐ No response

9. What is the highest level of education achieved by any member of your household?

- ☐ No education

- [ ] Some primary
- [ ] Complete primary
- [ ] Some secondary
- [ ] Complete secondary
- [ ] College education
- [ ] Higher
- [ ] No response

10. Religion

- [ ] Christian
- [ ] Muslim
- [ ] None
- [ ] Other: describe

11. Does your household currently own any dogs? Yes ☐ No ☐

12. Do you keep other animals in your household?

- [ ] Cattle
- [ ] Goats
- [ ] Sheep
- [ ] Pigs
- [ ] Poultry
- [ ] Cats
- [ ] Other: describe

Dog ownership

1. Number of adults and puppies currently in the household:

   Adults (over 3 months old):

   Puppies (under 3 months old):

2. Did you give away or sold adult dogs in the last year (how many)?

- ☐ Yes:
- ☐ No

3. Where these dogs vaccinated against rabies when they left the house? Give the number of them that were vaccinated.

- ☐ Yes:
- ☐ No

4. Without considering puppies, where are those dogs now?

- ☐ Another place in this ward
- ☐ Another ward in Blantyre city
- ☐ Blantyre rural
- ☐ Another district in Malawi
- ☐ Outside Malawi

5. What is your role in the care of the dogs in your household?

- ☐ I am the primary caretaker for all the dogs
- ☐ I am the primary caretaker for some of the dogs
- ☐ I sometimes provide care for the dogs
- ☐ I do not provide any care for the dogs in my household
- ☐ No response

6. What level of care does your household provide for your dogs?

- ☐ None
- ☐ Food
- ☐ Water
- ☐ Shelter
- ☐ Veterinary care
- ☐ Other: describe
- ☐ No response

7. Did you own any dog that died in the last year? (not including puppies)

☐ Yes ☐ No

8. How many dogs died?

Question per individual dog: General information

Dog ID (01,02, etc): Collar ID: Dog name:

1. Enter the dog's age in years and months:
2. Sex:

☐ Male ☐ Female

3. Dog breed:

☐ Local ☐ Other (please, specify):

4. Dog size:

☐ Small ☐ Medium ☐ Large

5. Body condition score (WSAVA score)

☐ 1-3 ☐ 4-5 ☐ 6 or more

6. Was this dog born from a dog that you own?

- [ ] It was a puppy from a dog in this house
- [ ] Got from somewhere else
- [ ] I don't know

If got somewhere else, how did you get this dog?

- [ ] Bought: [ ] from neighbour/friend [ ] from roadside seller [ ] from a shop
- [ ] Gift/adopted: [ ] from neighbour/friend [ ] roadside seller [ ] from a shop
- [ ] Found as stray

Where did the dog come from?

- [ ] This ward in Blantyre
- [ ] Another ward in Blantyre:
- [ ] In Blantyre rural:
- [ ] Another district in Malawi
- [ ] Outside Malawi:
- [ ] Unknown

7. What is the primary reason you own this dog?

- [ ] Security (guard dog)
- [ ] Companionship
- [ ] Hunting
- [ ] Breeding
- [ ] Wealth asset
- [ ] Other (describe)

8. How often do you feed your dog?

- ☐ Everyday
- ☐ Several times per week
- ☐ Several times a month
- ☐ Never
- ☐ Other: specify
- ☐ I don´t know

9. What is the main source of food for your dog?

- ☐ Pet food
- ☐ Kitchen leftovers
- ☐ Food prepared for the dog
- ☐ Other: specify

Question per individual dog: Reproduction information

1. Is your dog castrated/spayed?

☐ Yes ☐ No ☐ I don't know

2. How was your dog castrated/spayed?

- ☐ At a veterinary clinic
- ☐ During a sterilization campaign
- ☐ I don't know
- ☐ Other: specify

Question per individual dog: Dog management

1. Is your dog confined during the day?

☐ Always confined ☐ Sometimes free ☐ Always free

2. Is your dog confined during the night?

☐ Always confined ☐ Sometimes free ☐ Always free

3. Did your dog ever escape its confinement?

☐ Yes ☐ No ☐ Don't know ☐ Always free

Question per individual dog: Vaccinations

1. Total number of rabies vaccinations during its lifetime:
2. Was this dog vaccinated against rabies in the past year?

☐ Yes ☐ No

3. Who vaccinated the dog?

☐ Private vet
☐ AVO
☐ Mission rabies
☐ BSPCA
☐ I don't know

4. Is the dog vaccinated against other diseases?

☐ Yes (specify) ☐ No ☐ I don't know

Question per individual dog: Movements

1. How often do you take this dog outside of Malawi?

☐ Never
☐ Yearly
☐ Monthly
☐ Weekly
☐ Daily

2. How often do you take this dog to another district?

- ☐ Never
- ☐ Yearly
- ☐ Monthly
- ☐ Weekly
- ☐ Daily

3. How often do you take this dog to Blantyre rural?

- ☐ Never
- ☐ Yearly
- ☐ Monthly
- ☐ Weekly
- ☐ Daily

4. How often do you take this dog to another ward in Blantyre city?

- ☐ Never
- ☐ Yearly
- ☐ Monthly
- ☐ Weekly
- ☐ Daily

Thank you for completing the survey. Please click here to enter any other comments or relevant information from the respondent or problems encountered during the survey itself:
